# Proposed pathway for patients undergoing enhanced recovery after spinal surgery: protocol for a systematic review

**DOI:** 10.1186/s13643-020-1283-2

**Published:** 2020-02-21

**Authors:** Ana Licina, Andrew Silvers, Harry Laughlin, Jeremy Russell, Crispin Wan

**Affiliations:** 1 Austin Health, 145 Studley Road, Heidelberg, Victoria 3084 Australia; 2 Monash Health, 246 Clayton Road, Clayton, Australia; 3 St Vincent’s Health, 41 Victoria Parade, Fitzroy, Victoria 3065 Australia

**Keywords:** Enhanced recovery, Spinal surgery, Perioperative outcomes, Perioperative pathway, Preadmission processes, Prehabilitation, Perioperative nutrition, Minimally invasive surgery, Multimodal analgesia

## Abstract

**Background:**

The best evidence-enhanced recovery care pathway is yet to be defined for patients undergoing spinal surgery. Minimally invasive surgery, multimodal analgesia, early mobilization, and early postoperative nutrition have been considered as critical components of enhanced recovery in spinal surgery (ERSS). The objective of this study will be to synthesize the evidence underpinning individual components of a proposed multidisciplinary enhanced recovery pathway for patients undergoing spinal surgery.

**Methods:**

This is the study protocol for a systematic review of complex interventions. Our team identified 22 individual care components of a proposed pathway based on clinical practice guidelines and published reviews. We will include systematic reviews and meta-analysis, randomized controlled trials, non-randomized controlled trials, and observational studies in adults or pediatric patients evaluating any one of the pre-determined care components. Our primary outcomes will be all-cause mortality, morbidity outcomes (e.g., pulmonary, cardiac, renal, surgical complications), patient-reported outcomes and experiences (e.g., pain, quality of care experience), and health services outcomes (e.g., length of stay and costs). We will search the following databases (1990 onwards) MEDLINE, EMBASE, and Cochrane Library (Cochrane Database of Systematic Reviews and CENTRAL). Two reviewers will independently screen all citations, full-text articles, and abstract data. Potential conflicts will be resolved through discussion. The risk of bias for individual studies will be appraised using appropriate tools. A narrative synthesis will be provided with the information presented in the text and tables to summarize and explain the characteristics and findings of the included studies. Due to clinical and methodological heterogeneity, we do not anticipate to conduct meta-analyses. Confidence in cumulative evidence for each component of care will be classified according to the GRADE system.

**Discussion:**

This systematic review will identify, evaluate, and integrate the evidence underpinning individual components of a pathway for patients undergoing spinal surgery. The formation of an evidence-based pathway will allow for the standardization of clinical care delivery within the context of enhanced recovery in spinal surgery.

**Systematic review registration:**

PROSPERO CRD42019135289

## Background

Protocols and pathways in the field of enhanced recovery after surgery (ERAS) have focused on providing cohesive care bundles through improving the impact of individual components. Significant quality improvements in patient care have been shown during the application of ERAS in colorectal surgery [1]. A recent review of the effectiveness of the ERAS pathways in the UK concluded there was significant evidence that the programs could reduce the length of hospital stay without increasing readmissions [2]. There have been guidelines issued for multiple surgical subspecialties including colorectal, thoracic, gynecological, urological, and major arthroplasty surgery [3–10]. There is increasing research into the benefit of individual enhanced recovery elements and integrative protocols in spinal surgery. Prior reviews have explored the evidence base in patients undergoing spinal surgery. Broad concept recommendations have been made for the incorporation of individual components into an overarching program [8, 11]. For example, a 2019 review of studies investigating the application of formal enhanced recovery in spinal surgery (ERSS) protocols performed a qualitative analysis of outcomes such as length of stay, associated cost, and time to ambulation. It was concluded that ERSS is feasible, associated with shorter length of stay as well as earlier return to function [8]. This benefit was observed across several procedures and patient cohorts undergoing spinal surgery. Proposed enhanced recovery pathways in spinal surgery differ substantially across the incorporated elements. The inclusion of various technical elements in the individual programs is often based on the perceived clinical benefit obtained from each item, rather than an evidence-based approach.

To our knowledge, there is a gap with regard to identifying and defining the most efficacious elements of ERSS. Formulating an accurate protocol inclusive of multidisciplinary elements would allow for greater standardization of clinical care, with a stronger impact of enhanced recovery in spinal surgery. Qualitative assessment of the strength of the evidence underpinning each technical element has not been performed. Determination of clinical efficacy has not been performed for each of the individual elements. Consistency with regard to best multidimensional practice in ERSS would allow for standardization of care pathways and assist audit uniformity.

### Description of the pathway

Enhanced recovery pathways of other surgical subspecialties consist of five pillars of perioperative patient care:

- Preadmission period Preparatory time frame during which patients can be assessed, optimized and counseled
- Preoperative period Time period from hospital admission to the commencement of surgery
- Intraoperative period Time period during the surgical and anesthetic care
- Postoperative period Time frame following surgical intervention during which recovery is attained and processes instigated in order to return to optimal level of function
- Audit and compliance processes Systematic approach to peer-reviewed quality of care ensuring quality assurance and improvement of personnel, processes, and outcomes

Pathway components are modified according to the clinical need of the surgical specialty. For example, in colorectal care, particular attention is paid to the utility of bowel preparation, early feeding processes, and perioperative fluid management [7]. The 2019 ERAS protocol for thoracic surgery considered perioperative analgesia and minimally invasive surgery critical components of the pathway [3]. For the enhanced recovery in spinal surgery, previous systematic reviews of proposed interventions have been published [8, 11, 12]. The critical components of the pathway in spinal surgery are considered to be the provision of comprehensive perioperative nutrition, multimodal analgesia, minimally invasive surgery where clinically feasible, and early mobilization [11]. The panel of authors considered the proposed pathway within the five perioperative pillars, whilst incorporating the spinal surgery-specific critical components. We have identified 22 components of the proposed pathway (see Additional files 1, 2, and 3 for further details). We identified these components based on the enhanced recovery protocols in other surgical subspecialties and prior qualitative reviews of ERAS in spinal surgery (Table 1).

**Table 1:** Individual components of enhanced recovery pathway in spinal surgery, grouped according to care delivery episode

Preadmission period	Intraoperative period	Postoperative period
1. Preadmission information, education and counseling	8. Perioperative blood conservation strategies	17. Thromboprophylaxis
2. Risk assessment, preoperative optimization, including lifestyle factor modification	9. Minimally invasive surgical approaches	18. Urinary drainage
2.1 Preoperative risk stratification	10. Skin preparation and antimicrobial prophylaxis	19. Postoperative nutrition and fluid management
2.2 Preoperative optimization	11. Local anesthetic infiltration	20. Postoperative glycemic control
2.3 Alcohol use	12. Anesthetic protocol	21. Early mobilization
2.4 Tobacco use	13. Prevention of post-operative nausea and vomiting	22. Audit
3. Prehabilitation	14. Maintenance of normothermia	
4. Pre-operative nutritional care	15. Fluid management	
4.1 Pre-operative nutritional screening	16. Perioperative analgesic measures	
4.2 Pre- and peri-operative immune-nutrition		
5. Management of anemia		
Preoperative period		
6. Preoperative fasting and carbohydrate loading		
7. Pre-emptive analgesia		

### Why is it important to do this review?

Preoperative, preadmission, intraoperative, and postoperative processes are a continuum of perioperative care received by each patient. Prior ERSS pathway proposals have laid the foundations for the development of more comprehensive approaches. There is a demonstrable variation in practice in the composition and application of enhanced recovery pathways in spinal surgery. There is a clinical need to systematically review a more comprehensive combination of individual components of an ERSS pathway. Combining the individual elements together in a consistent pathway has been shown to result in incremental improvements in patient care outcomes [13, 14]. Greater standardization of ERSS pathways would allow for increased inter-study validity and reliability in performing comparative research. An evidence-based ERSS pathway would assist decision-makers with institutional adoption and implementation.

### Aims and objectives

The aim of this complex intervention systematic review is to propose an evidence-based integrated enhanced recovery pathway specific to patients undergoing spinal surgery. We have identified 22 individual elements of enhanced recovery in spinal surgery in line with other standardized ERAS pathways and expert panel assessment [3–8, 15–18] (see Additional file 1).

1. We aim to conduct a systematic review of evidence underpinning each pre-defined component of the proposed pathway. Each component may have been studied individually or within the context of an enhanced recovery pathway.
2. We aim to create an evidence-based assessment of the strength of the literature detected for each pre-defined component of the proposed pathway.

## Methods/design

The present review protocol is being reported in accordance with the reporting guidelines, the Preferred Reporting Items for Systematic Reviews and Meta-Analyses Protocol (PRISMA-P) statement (see PRISMA-P checklist in Additional file 2) [19]. This review protocol was registered within the International Prospective Register of Systematic Reviews (PROSPERO) (registration number CRD42019135289) [20].

The authors identified and defined the essential components of enhanced recovery within the area of spinal surgery. The authors performed this process by reviewing the current externally validated enhanced recovery protocols as recommended by the ERAS Society. We compared and contrasted the current published guidelines and reviews for gastrointestinal surgery, thoracic surgery, orthopedic surgery, and gynecological surgery [3–8, 12]. We identified and applied the relevant components to the field of spinal surgery. In addition, we compared and contrasted the technical elements present in the published prior reviews on enhanced recovery in spinal surgery [8, 11, 12]. A brief summary of this process has been provided in Additional file 1.

Our review protocol falls within the framework and definition of a complex systematic review due to the underlying intervention complexity and intertwined causal pathways. The planned review targets multiple groups of participants and will be applicable at multiple organizational levels and will require multifaceted adoption. It will need to be applied in a dynamic multidimensional environment. The planned review will therefore be performed according to the methodological standards for complex reviews [21–28].

### Eligibility criteria

Studies will be selected according to the following criteria below (and a summary in Table 2).

**Table 2:** Spinal surgical procedures

Anterior cervical decompression and fusion	
Posterior cervical decompression/fusion	
Thoracic decompression and fusion	
Scoliosis correction	
Multilevel decompression and fusion	
Lumbar decompression and fusion	
Lumbar laminectomy	
Lumbar micro-discectomy	

### Types of studies

We will include systematic reviews and meta-analysis, randomized controlled trials, non-randomized controlled studies, and observational studies (e.g., cohort studies, case-control studies, cross-sectional studies, and case series).

We will include studies published in the English language. Due to the need for a contemporaneous review, studies published prior to 1990 will be excluded. Studies will not be excluded based on the publication status.

### Types of participants

We will include all adult and pediatric population undergoing spinal surgical procedures (Table 2). We will include patients undergoing spinal surgical procedure on any anatomical site including cervical (anterior or posterior cervical decompression and fusion), thoracic (e.g., thoracic decompression and fusion), lumbar (e.g., lumbar decompression and fusion, lumbar laminectomy, lumbar micro-discectomy), sacral (e.g., scoliosis correction), or any combination of these.

### Types of interventions

The interventions of interest have been classified in 5 perioperative pillars: preadmission period, preoperative period, intraoperative period, postoperative period, and audit and compliance processes. The interventions have been defined as the pre-determined components of the enhanced recovery pathway as applicable to spinal surgery (Table 1). The interventions may have been studied alone or in any one combination.

### Types of outcome measures

One of the hallmarks of enhanced recovery reviews is the heterogeneity of outcomes due to the depth and breadth of the patient care received. Our primary outcomes will be all-cause mortality, morbidity outcomes (e.g., pulmonary, cardiac, renal, surgical complications), patient-reported outcomes and experiences (e.g., pain, quality of care experience), and health services outcomes (e.g., length of stay and costs) (see Table 3 and the “Outcomes and prioritization” section below).

**Table 3:** Summary of eligibility criteria

Study characteristic	Inclusion criteria	Exclusion criteria
Patient population	Adults undergoing spinal surgical procedures. Pediatric population undergoing spinal surgical procedures.	Patients undergoing non-surgical management of spinal conditions Traumatic patients without surgery
Intervention-treatment	Twenty-two pre-defined components of an ERSS pathway (as outlined in Table 1) alone or in combination with another component Other proposed ERSS pathways incorporating one or more pre-defined interventions will be included.	
Comparator	Standard of care, no treatment, or placebo	
Outcomes	Mortality from all causes Morbidity including: pulmonary, cardiac and renal complication rates, surgical complication rates (including readmissions) Patient reported experiences and outcomes (PROMs/PREMS): pain-related outcomes (e.g., pain score rating, pain management satisfaction), quality of care (readiness for surgery, quality of care patient scores, quality of recovery after surgery), Health service-related outcomes: length of stay (in hospital, in ICU) and economic/financial outcomes (e.g., costs of patient stay)	
Study design	Systematic reviews, meta-analysis Randomized controlled trials Non-randomized studies Observational studies (cohort studies, case-control studies, cross-sectional studies, case series)	Case reports
Study setting	Inpatient care (including patients whose condition requires admission to a hospital same-day discharge surgical)	Outpatient clinics, medical, and non-surgical management of spinal conditions
Timing	Perioperative process-preadmission, preoperative, intraoperative, and postoperative setting	Studies incorporating long-term (greater than three months) postoperative rehabilitation

### Information sources and search strategy

We will search the following electronic databases (from 1990 onwards): MEDLINE via Ovid SP; EMBASE via Ovid SP; and Cochrane Library (Cochrane Database of Systematic Reviews and CENTRAL). In addition, the authors will search the grey literature through the following specific search engines: Google Scholar, OpenGrey, and GreyNet [29–31]. For the search strategy, we will combine keyword and subject headings in combination with validated filters in each of the pre-determined electronic databases [28]. The draft search strategy specific to MEDLINE is included in Additional file 3.

### Data selection and screening process

All articles identified from the literature search will be screened by two reviewers independently using an electronic screening form (Covidence web platform: http://www.COVIDENCE.org ). First, titles and abstracts of articles returned from initial searches will be screened based on the eligibility criteria outlined above. Second, full texts will be examined in detail and screened for eligibility. Third, references of all considered articles will be hand-searched to identify any relevant report missed in the search strategy. Any disagreements will be resolved by discussion to meet a consensus, if necessary.

A PRISMA flow diagram showing details of studies included and excluded at each stage of the study selection process will be provided.

Study information will be stored and managed using Endnote X9 throughout the review process.

### Data collection

A data extraction form will be designed and used to extract equivalent information from each study. Information of interest will include the following:

- Study characteristics: study design, year of publication, journal, year (or period) of study conduct, sample size, setting, and other fields to capture data relevant to the assessment of study methodological quality (see the “Risk of bias assessment” subsection).
- Participant characteristics: population sampled, age (e.g., mean with standard deviation, range) and gender (e.g., percentage of female participants), type of spinal surgery.
- Intervention (individual components of the pathway) and comparator characteristics and definitions.
- Outcome results: definitions and assessment tools.

Data extraction forms will be piloted initially on a small number of included studies. Subsequently, each of the included studies will be abstracted by two team members, independently, and potential conflicts will be resolved through discussion. Authors of primary publications will be contacted for data clarifications or missing outcome data, as necessary.

### Outcomes and prioritization

We defined our primary outcomes in terms of the following groups:

- Mortality from all causes.
- Morbidity, including pulmonary, cardiac, and renal complication rates; surgical complication rates; and readmission rates.
- Patient-reported experiences and outcomes (PREMs/PROMs), including pain-related outcomes (pain score rating, pain management satisfaction), readiness for surgery, quality of care patient scores, and quality of recovery outcomes.
- Health service-related outcomes, including length of stay and reported economic/financial outcomes (e.g., costs of the length of stay).

Our chosen outcome measures although grouped in several primary headings are broad. This is due to the anticipated heterogeneity of the reporting for the pre-specified components of the pathway. Our approach to the outcome definition and synthesis is in line with other reported enhanced recovery specialty pathways. Outcomes analyzed in previous reviews consisted of a length of stay, readmission, complication rate, and patient satisfaction measures [3, 4, 7–10, 32].

### Risk of bias in individual studies

Risk of bias in randomized controlled studies will be assessed using the Cochrane Risk of Bias tool [33]. This tool considers several domains of bias: randomization, allocation concealment, blinding, accounting of patients and outcome events, and selective outcome reporting bias [34]. Risk of bias appraisal in non-randomized and observational studies can introduce difficulty due to a lack of a generic robust bias assessment tool [35]. We will use the ROBINS-I (risk of bias in non-randomized studies of interventions) to assess the risk of bias in non-randomized studies [36]. ROBINS-I is the preferred tool to be used in Cochrane Reviews for non-randomized studies of interventions. The authors of the GRADE working group have recently issued recommendations on how the ROBINS-I tool should be used within the context of the GRADE guidelines [37]. In order to assess the risk of bias in systematic reviews, we will use the revised AMSTAR-2 tool [38]. We will use the risk of bias assessment in individual studies to inform our assessment of study limitations across the body of evidence.

### Data synthesis

We will perform qualitative (narrative) synthesis of included studies. We will perform this process through thematic synthesis for each proposed component of the complex intervention pathway [26]. We plan to analyze, synthesize, and grade the quality of the body of evidence across outcomes with regard to individual pathway components [39]. In the first stage of the synthesis, we will systematically describe the data obtained from each study and its findings. In the second stage, we will group the obtained data under the relevant outcome and individual pathway element. The endpoint of the GRADE evidence summary will consist of the Evidence Profile (EP) [34]. This thematic narrative synthesis will not provide a single estimate measure for each outcome of each pathway component. Summary of findings table will not be constructed [40]. Narrative analysis of each pathway component will present the summarized data used to inform the synthesis in the form of a forest plot [27]. Forest plot will be used in order to summarize data for each relevant outcome of each pathway component. Finally, we will perform a narrative analysis of the obtained data pertaining to each pathway component. For each component of the pathway, we will assess clinical diversity across studies by examining the variability in the intervention used, the type of surgery and the participant group. We have chosen this method in order to answer the key question of “what happens when the complex intervention is implemented?”. This method of evidence synthesis is in line with other published enhanced recovery reviews [3, 5, 7, 15, 16, 41, 42]. Meta-analyses will not be conducted due to the anticipated inherent methodological heterogeneity of the studies with regard to design, populations, procedures, and outcomes [43]. As such, it is unlikely that quantitative synthesis would be meaningful in this review of complex intervention [27].

### Meta-biases

Selective reporting of outcomes within studies will be reviewed by comparison with published protocols, review of study registration information on appropriate sources (e.g., trial registration websites, PROSPERO for systematic reviews) [28].

### Confidence in cumulative evidence

We will use the GRADE Handbook to guide the process of rating the evidence. In the context of a systematic review, the ratings of the quality of evidence reflect the extent of our confidence that the estimates of the effect are correct [39]. Quality of evidence will be classified according to the Grading of Recommendations, Assessment, Development and Evaluation (GRADE) system into one of four categories: *high, moderate, low, and very low* [40]. Grading of the quality of evidence according to this system will be performed for pertinent study outcomes classified by the individual elements of the pathway as outlined in Table 1. A recommended strategy for systematic review authors is to use this approach in order to rate the quality of evidence for each outcome across studies, i.e., for a body of evidence [38, 39]. Quality of the body of evidence will be assessed according to study limitations, imprecision, the inconsistency of results, indirectness of evidence, and publication bias [38]. Evidence based on randomized controlled trials will be considered as high quality unless confidence in the evidence is decreased due to study limitations, the inconsistency of results, indirectness of evidence, imprecision, and reporting biases. Observational studies will be considered low quality; however, they may be graded higher if the treatment effect observed is very large or if there is evidence of a dose-response relationship [39, 40]. Quality of evidence can be seen as occurring in a continuum; therefore, any discrete categorization of continuous evidence base does involve a degree of arbitrariness. As we are undertaking a systematic review, rather than developing a guideline, we will not be making recommendations on the utility of the pathway components [27, 40].

## Discussion

Interest in the delivery of enhanced recovery pathways within the context of spinal surgery has grown substantially in the last decade. Many of the studies illustrating the institutional implementation of some components of ERSS have shown a significant reduction in the length of stay and consequent improved financial outcome [44, 45]. Effective interventions not only provide for better outcomes for individual patients, but for more efficient use of resources in healthcare and avoidance of ineffective high-cost interventions [46]. To date, there has been no review of the merits of individual pathway elements in enhanced recovery in spinal surgery protocols. An evidence-based review of the individual components delivering a proposed pathway of enhanced recovery in spinal surgery is timely. We have proposed a multidisciplinary pathway based on the current best evidence in spinal surgery and ERAS society resources [3–8, 47]. Prior proposed pathways have often concentrated on components critical to spinal surgery including minimally invasive surgery, multimodal analgesia, early mobility and perioperative nutrition. Although, these are core principles of an enhanced recovery protocol in spinal surgery, incremental benefit is obtained through a more comprehensive pathway. Through this additive effect, better outcomes of patient care may be achievable.

We will synthesize the best available evidence for each strategically chosen individual component of the pathway. We will review the studies of individual components delivered outside as well as a part of enhanced recovery protocols in chosen pathways. We have chosen this approach in order to have the best available evidence for each individual presented component. We have hypothesized that the most effective care pathway in its totality is one with the best evidence for the delivery of individual components.

Enhanced recovery concepts have only recently been introduced in neurosurgery. As such, we anticipate a limited availability of methodologically highly ranked studies specific to this area of practice.

The methodological limitations of this study include the need for the assessment of observational studies during the review process. It is unlikely that our research question can be answered through a review of randomized controlled trials and meta-analysis only. Observational studies will be rated as low-quality evidence unless they are significantly sized with clear pre-defined outcomes. This does expose our study to methodological biases—including selection and retrospective biases [35]. When feasible, we will evaluate the published protocol for studies, thereby assessing for reported outcomes. We will assess the risk of bias at the study level in both randomized and non-randomized studies. As per the authors of the GRADE system, there is a possibility of misuse of the ROBINS-I tool when used within the confines of rating quality of evidence [37]. There is a risk of inappropriate rating up of non-randomized studies, which would normally be considered low-quality evidence

We will disseminate the results of this review in a peer-reviewed academic journal. We expect our results will have important implications for the care of patients undergoing spinal surgery within the context of enhanced recovery. Our results will assist institutional decision makers in adopting and adapting suitable enhanced recovery in spinal surgery pathways.

## Supplementary information

**Additional file 1.** Individual components of the pathway for patients undergoing enhanced recovery after spinal surgery

**Additional file 2.** PRISMA-P checklist

**Additional file 3.** Draft search strategy

## Data Availability

Not applicable

## Abbreviations

ERAS: Enhanced recovery after surgery
ERSS: Enhanced recovery after spinal surgery
GRADE: Grading of Recommendations, Assessment, Development and Evaluation
PRISMA: Preferred Reporting Items for Systematic Reviews and Meta-Analysis
