# Supplementary material for: Proposed pathway for patients undergoing enhanced recovery after spinal surgery: protocol for a systematic review
Source: Syst Rev. 2020 Feb 21;9:39. doi: 10.1186/s13643-020-1283-2 (PMC7035675; doi:10.1186/s13643-020-1283-2)
Supplement: Supplementary file 1 — Additional file 1. Individual components of the pathway for patients undergoing enhanced recovery after spinal surgery [file 13643_2020_1283_MOESM1_ESM.docx]

**Additional file 1.** **Individual components of the pathway for patients undergoing enhanced recovery after spinal surgery**

We identified all relevant ERAS clinical guidelines available through the ERAS society and disseminated widely (1-18). In addition, a thematic synthesis of all available components was performed. Briefly, we identified **twenty components** of the standardized recovery pathways across surgical specialties as outlined in the *Table 1a* (below).

*Additional Table 1a.*

*Components of the pathway identified through search of the literature*

| *Preadmission Period* | *Intraoperative Period* | *Postoperative Period* |
| --- | --- | --- |
| 1. Preadmission information, education and counseling |  | 17. Thromboprophylaxis |
| 2. Risk assessment, preoperative optimization, including lifestyle factor modification | 9. Minimally invasive surgical approaches | 18. Urinary drainage |
| 2.1 Preoperative risk stratification | 10. Skin preparation and antimicrobial prophylaxis | 19. Postoperative nutrition and fluid management |
| 2.2 Preoperative optimization |  | 20. Postoperative glycemic control |
| 2.3 Alcohol use | 12. Anaesthetic protocol | 21. Early mobilization |
| 2.4 Tobacco use | 13. Prevention of post-operative nausea and vomiting | 22. Audit |
| 3. Prehabilitation | 14. Avoiding hypothermia |  |
| 4. Pre-operative nutritional care | 15. Fluid management |  |
| 4.1 Pre-operative nutritional screening | 16. Perioperative analgesic measures |  |
| 4.2 Pre- and peri-operative immune-nutrition |  |  |
| 5. Management of anaemia |  |  |
| *Preoperative Period* |  |  |
| 6. Preoperative fasting and carbohydrate loading |  |  |
| 7.Pre-emptive analgesia |  |  |

Subsequently, the authors sought to identify pertinent systematic reviews or meta-analysis specific to spinal surgery. We performed a literature search in order to identify existing evidence base underpinning enhanced recovery components in spinal surgery. We performed this through a MEDLINE via OVIDsp database using keywords relevant to enhanced recovery in spinal surgery. Our search strategy was equivalent to the proposed review search strategy as available in Additional files. We used the review filter to access the relevant studies included here. We subsequently performed a thematic synthesis of the qualitative reviews in spinal surgery outlining pathway components in enhanced recovery in spinal surgery (*see results in Table 2a*). We noted congruence of **twenty common components** in published reviews between spinal surgery enhanced recovery elements and other surgical specialties (19-21). Two additional components, *“perioperative blood conservation strategies”* and *“local anaesthetic infiltration”,* were included in the proposed pathway for the planned systematic review.

*Additional Table 2a. Reviews in enhanced recovery in spinal surgery used for informing the selection of individual components of the pathway.*

| Author and year | Title | Description | Synopsis of findings |
| --- | --- | --- | --- |
| Elsarrag et al,  2019 | Enhanced recovery after spine surgery: a systematic review | Qualitative literature review of ERAS programs in spinal surgery | Introductory studies demonstrated the ability to reduce length of stay, accelerate return of function, minimize postoperative pain and save costs; |
| Corniola et al,  2019 | Enhanced recovery after spine surgery: review of literature | Qualitative literature review of spinal surgery studies applying aspects of ERAS | Qualitative synthesis of evidence with a focus on the benefits of Minimally Invasive Spinal Surgery |
| Dietz et al, 2019 | Enhanced recovery after surgery (ERAS) for Spine Surgery: A Systematic review | Systematic review of published enhanced recovery studies in spinals surgery | Qualitative synthesis and tabulation of evidence;  Finding of pre-operative analgesic benefit in spinal surgery |
| Wainwright et al,  2016 | Enhanced recovery after surgery and its applicability for major spine surgery | Qualitative literature review and analysis of individual components applicable to ERSS | Individual components recommended included:  -Preoperative education  -Multimodal pain management  -minimally invasive surgical approaches  -Management of blood loss  -Management of nutrition  -Physiotherapy |

For further clarifications and details on the process, please contact the corresponding author.

Supplementary references:

1. Abola RE, Bennett-Guerrero E, Kent ML, Feldman LS, Fiore JF, Jr., Shaw AD, et al. American Society for Enhanced Recovery and Perioperative Quality Initiative Joint Consensus Statement on Patient-Reported Outcomes in an Enhanced Recovery Pathway. Anesth Analg. 2018;126(6):1874-82.

2. Adamina M, Kehlet H, Tomlinson GA, Senagore AJ, Delaney CP. Enhanced recovery pathways optimize health outcomes and resource utilization: a meta-analysis of randomized controlled trials in colorectal surgery. Surgery. 2011;149(6):830-40.

3. Azhar RA, Bochner B, Catto J, Goh AC, Kelly J, Patel HD, et al. Enhanced Recovery after Urological Surgery: A Contemporary Systematic Review of Outcomes, Key Elements, and Research Needs. European urology. 2016;70(1):176-87.

4. Cerantola Y, Valerio M, Persson B, Jichlinski P, Ljungqvist O, Hubner M, et al. Guidelines for perioperative care after radical cystectomy for bladder cancer: Enhanced Recovery After Surgery (ERAS((R))) society recommendations. Clinical nutrition (Edinburgh, Scotland). 2013;32(6):879-87.

5. Dietz N, Sharma M, Adams S, Alhourani A, Ugiliweneza B, Wang D, et al. Enhanced Recovery After Surgery (ERAS) for Spine Surgery: A Systematic Review. World neurosurgery. 2019;130:415-26.

6. Fearon KCH, Ljungqvist O, Von Meyenfeldt M, Revhaug A, Dejong CHC, Lassen K, et al. Enhanced recovery after surgery: A consensus review of clinical care for patients undergoing colonic resection. Clinical Nutrition. 2005;24(3):466-77.

7. Gotlib Conn L, Rotstein OD, Greco E, Tricco AC, Perrier L, Soobiah C, et al. Enhanced recovery after vascular surgery: protocol for a systematic review. Systematic reviews. 2012;1:52.

8. Hedrick TL, McEvoy MD, Mythen MG, Bergamaschi R, Gupta R, Holubar SD, et al. American Society for Enhanced Recovery and Perioperative Quality Initiative Joint Consensus Statement on Postoperative Gastrointestinal Dysfunction Within an Enhanced Recovery Pathway for Elective Colorectal Surgery. Anesthesia & Analgesia. 2018;126(6):1896-907.

9. Ljungqvist O, Scott M, Fearon KC. Enhanced Recovery After Surgery: A Review. JAMA surgery. 2017;152(3):292-8.

10. Markar SR, Karthikesalingam A, Low DE. Enhanced recovery pathways lead to an improvement in postoperative outcomes following esophagectomy: systematic review and pooled analysis. Diseases of the esophagus : official journal of the International Society for Diseases of the Esophagus. 2015;28(5):468-75.

11. Melloul E, Hubner M, Scott M, Snowden C, Prentis J, Dejong CH, et al. Guidelines for Perioperative Care for Liver Surgery: Enhanced Recovery After Surgery (ERAS) Society Recommendations. World journal of surgery. 2016;40(10):2425-40.

12. Moonesinghe SR, Grocott MPW, Bennett-Guerrero E, Bergamaschi R, Gottumukkala V, Hopkins TJ, et al. American Society for Enhanced Recovery (ASER) and Perioperative Quality Initiative (POQI) joint consensus statement on measurement to maintain and improve quality of enhanced recovery pathways for elective colorectal surgery. Perioper Med (Lond). 2017;6:6-.

13. Paton F, Chambers D, Wilson P, Eastwood A, Craig D, Fox D, et al. Effectiveness and implementation of enhanced recovery after surgery programmes: a rapid evidence synthesis. BMJ Open. 2014;4(7):e005015-e.

14. Soffin EM, Gibbons MM, Ko CY, Kates SL, Wick E, Cannesson M, et al. Evidence Review Conducted for the Agency for Healthcare Research and Quality Safety Program for Improving Surgical Care and Recovery: Focus on Anesthesiology for Total Knee Arthroplasty. Anesth Analg. 2019;128(3):441-53.

15. Soffin EM, Gibbons MM, Ko CY, Kates SL, Wick EC, Cannesson M, et al. Evidence Review Conducted for the Agency for Healthcare Research and Quality Safety Program for Improving Surgical Care and Recovery: Focus on Anesthesiology for Total Hip Arthroplasty. Anesth Analg. 2019;128(3):454-65.

16. Soffin EM, Gibbons MM, Wick EC, Kates SL, Cannesson M, Scott MJ, et al. Evidence Review Conducted for the Agency for Healthcare Research and Quality Safety Program for Improving Surgical Care and Recovery: Focus on Anesthesiology for Hip Fracture Surgery. Anesth Analg. 2019;128(6):1107-17.

17. Soffin EM, Gibbons MM, Wick EC, Kates SL, Cannesson M, Scott MJ, et al. Evidence Review Conducted for the Agency for Healthcare Research and Quality Safety Program for Improving Surgical Care and Recovery: Focus on Anesthesiology for Hip Fracture Surgery. Anesth Analg. 2018.

18. Wischmeyer PE, Carli F, Evans DC, Guilbert S, Kozar R, Pryor A, et al. American Society for Enhanced Recovery and Perioperative Quality Initiative Joint Consensus Statement on Nutrition Screening and Therapy Within a Surgical Enhanced Recovery Pathway. Anesth Analg. 2018;126(6):1883-95.

19. Elsarrag M, Soldozy S, Patel P, Norat P, Sokolowski JD, Park MS, et al. Enhanced recovery after spine surgery: a systematic review. Neurosurg Focus. 2019;46(4):E3.

20. Corniola MV, Debono B, Joswig H, Lemée J-M, Tessitore E. Enhanced recovery after spine surgery: review of the literature. 2019;46(4):E2.

21. Wainwright TW, Immins T, Middleton RG. Enhanced recovery after surgery (ERAS) and its applicability for major spine surgery. Best Pract Res Clin Anaesthesiol. 2016;30(1):91-102.
