# Supplementary material for: Proposed pathway for patients undergoing enhanced recovery after spinal surgery: protocol for a systematic review
Source: Syst Rev. 2020 Feb 21;9:39. doi: 10.1186/s13643-020-1283-2 (PMC7035675; doi:10.1186/s13643-020-1283-2)
Supplement: Supplementary file 3 — Additional file 3. Draft search strategy [file 13643_2020_1283_MOESM3_ESM.docx]

Literature Search items

Database: Ovid Medline (R) 1990- October 2019

| 1. Neurosurgical Procedures.mp or exp Neuro Surgical Procedures/ |
| --- |
| 2. Spinal Surgery, Cervical.mp. |
| 3. Spinal Surgery, Thoracic.mp. |
| 4. Spinal Surgery Lumbar.mp. |
| 5. Microdiscectomy.mp. |
| 6. Laminectomy.mp. |
| 7. Fusion.mp. |
| 8. Cervical decompression.mp. |
| 9. Scolioisis.mp. |
|  |
| 10. ( spin$ surg$).tw. |

| 11. or/1-10 |
| --- |
| 12.Chewing Gum/[Enhanced Recovery] |
| 13.Early Ambulation/ |
| 14.Exercise Therapy/ |
| 15. Heating/ |
| 16. Intraoperative Care/mt |
| 17. Preoperative Care/mt |
| 18. Perioperative Care/mt |
| 19.Postoperative Care/mt |
| 20. Patient Education as Topic/ |
| 21.Surgical Procedures, Minimally Invasive/ |
| 22. exp Anesthesia/ |
| 23. an?esthesia.tw. |
| 24. an?esthetic?.tw. |
| 25. (accelerat$ adj2 mobil$).tw. |
| 26. (accelerat$ adj2 ambulat$).tw. |
| 27. (accelerat$ adj2 walk$).tw.  28.(accelerat$ adj2 eat$).tw.  29.(accelerat$ adj2 rehab$).tw.  30.(chew$ adj1 gum?).tw.  31.(client$ adj educat$).tw.  32.(client$ adj teach$).tw.  33.(client$ adj counsel$).tw.  34.(client$ adj expectation$).tw.  35.(crystalloid adj manage$).tw.  36.(crystalloid adj admin$).tw.  37.(earl$ adj2 mobil$).tw.  38.(earl$ adj2 ambulat$).tw.  39.(earl$ adj2 walk$).tw.  40.(earl$ adj2 feed$).tw.  41.(earl$ adj2 nutrition$).tw.  42.(earl$ adj2 eat$).tw.  43.(earl$ adj2 rehab$).tw.  44.(enhanced adj recover$).tw.  45.ERAS.tw.  46.(fast adj tract$).tw.  47.(fast adj track$).tw.  48.(heat$ adj2 patient$).tw.  49.intraoperative.mp. and (intravenous adj fluid?).tw.  50.intraoperative.mp. and (IV adj fluid?).tw.  51.intraoperative.mp. and Infusions, Intravenous/  52.intraoperative.mp. and Intubation, Gastrointestinal/  53.intraoperative.mp. and (NG adj tube?).tw.  54.intraoperative.mp. and (nasogastric adj tube?).tw.  55.intraoperative.mp. and (fluid? adj1 restrict$).tw.  56.intraoperative.mp. and (abdominal adj drain$).tw.  57.intraoperative.mp. and Anti-Inflammatory Agents, Non-Steroidal/  58.intraoperative.mp. and NSAID?.tw.  59.(intraoperative and analgesi$).mp.  60.(intraoperative and epidural?).mp.  61.(intraoperative and narcotic?).mp.  62.(intraoperative and (fluid adj therap$)).mp.  63.intraoperative.mp. and (fluid adj manag$).tw.  64.intraoperative.mp. and (electrolyte$ adj manag$).tw.  65.(intraoperative and (pain adj manage$)).mp.  66.(intraoperative and (vein adj thrombos?s)).mp.  67.((intraoperative adj care) and enhanced).tw.  68.((intraoperative adj care) and accelerat$).tw.  69.((intraoperative adj care) and early).tw.  70.intra-operative.mp. and (intravenous adj fluid?).tw.  71.intra-operative.mp. and (IV adj fluid?).tw.  72.intra-operative.mp. and Infusions, Intravenous/  73.intra-operative.mp. and Intubation, Gastrointestinal/  74.intra-operative.mp. and (NG adj tube?).tw.  75.(postoperative and (fluid adj therap$)).mp.  76.postoperative.mp. and (fluid adj manage$).tw.  77.postoperative.mp. and (electrolyte$ adj manag$).tw.  78.(postoperative and analgesi$).mp.  79.(postoperative and opioid?).mp.  80.(postoperative and opiat$).mp.  81.(postoperative and epidural?).mp.  82.postoperative.mp. and Anti-Inflammatory Agents, Non-Steroidal/  83.postoperative.mp. and NSAID?.tw.  84.(postoperative and (pain adj manage$)).mp.  85.postoperative.mp. and (care adj map?).tw.  86.postoperative.mp. and (care adj plan$).tw.  87.postoperative.mp. and (treatment adj plan$).tw.  88.(postoperative and (clinical adj path$)).mp.  89.(postoperative and (care adj path$)).tw.  90.(postoperative and (critical adj path$)).mp.  91.(postoperative and (case adj management)).mp.  92.(postoperative and (patient adj discharge)).mp.  93.(postoperative and (discharge adj plan$)).mp.  94.(postoperative and (vein adj thrombos?s)).mp.  95.(postoperative and antiemetic?).mp.  96.(postoperative and anti-emetic?).mp.  97.(postoperative and ileus).mp.  98.((postoperative adj care) and enhanced).tw.  99.((postoperative adj care) and accelerat$).tw.  100.((postoperative adj care) and early).tw.  101.post-operative.mp. and (regular adj diet?).tw.  102.post-operative.mp. and (normal adj diet?).tw.  103.post-operative.mp. and Enteral Nutrition/  104.(post-operative and catheter?).mp.  105.(post-operative and (fluid adj therap$)).mp.  106.post-operative.mp. and (fluid adj manage$).tw.  107.post-operative.mp. and (electrolyte$ adj manag$).tw.  108.(post-operative and analgesi$).mp.  109.(post-operative and opioid?).mp.  110.(post-operative and opiat$).mp.  111.(post-operative and epidural?).mp.  112.post-operative.mp. and Anti-Inflammatory Agents, Non-Steroidal/  113.post-operative.mp. and NSAID?.tw.  114.(post-operative and (pain adj manage$)).mp.  115.post-operative.mp. and (care adj map?).tw.  116.post-operative.mp. and (care adj plan$).tw.  117.post-operative.mp. and (treatment adj plan$).tw.  118.(post-operative and (clinical adj path$)).mp.  119.(post-operative and (care adj path$)).tw.  120.(post-operative and (critical adj path$)).mp.  121.(post-operative and (case adj management)).mp.  123.(post-operative and (patient adj discharge)).mp.  124.post-operative.mp. and (discharge adj plan$).tw.  125.(post-operative and (vein adj thrombos?s)).mp.  126.(post-operative and antiemetic?).mp.  127.(post-operative and anti-emetic?).mp.  128.(post-operative and ileus).mp.  129.((post-operative adj care) and enhanced).tw.  130.((post-operative adj care) and accelerat$).tw.  131.((post-operative adj care) and early).tw.  132.(preoperative and fasting).mp.  133.preoperative.mp. and Anti-Inflammatory Agents, Non-Steroidal/  134.preoperative.mp. and NSAID?.tw.  135.preoperative.mp. and carbohydrate$.tw.  136.(preoperative and probiotic?).mp.  137.(preoperative and pro-biotic?).mp.  138.preoperative.mp. and hydrat$.tw.  139.preoperative.mp. and dehydrat$.tw.  140.preoperative.mp. and de-hydrat$.tw.  141.preoperative.mp. and stress.tw.  142.((preoperative adj care) and enhanced).tw.  143.((preoperative adj care) and accelerat$).tw.  144.((preoperative adj care) and early).tw.  145.(pre-operative and fasting).mp.  146.pre-operative.mp. and Anti-Inflammatory Agents, Non-Steroidal/  147.pre-operative.mp. and NSAID?.tw.  148.pre-operative.mp. and carbohydrate$.tw.  149.(pre-operative and probiotic?).mp.  150.(pre-operative and pro-biotic?).mp.  151.pre-operative.mp. and hydrat$.tw.  152.pre-operative.mp. and dehydrat$.tw.  153.pre-operative.mp. and de-hydrat$.tw.  154.pre-operative.mp. and stress.tw.  155.((pre-operative adj care) and enhanced).tw.  156.((pre-operative adj care) and accelerat$).tw.  157.((pre-operative adj care) and early).tw.  158.(rapid$ adj2 recover$).tw.  159.(rapid adj2 mobil$).tw.  160.(rapid adj2 ambulat$).tw.  161.(rapid adj2 walk$).tw.  162.(rapid adj2 feed$).tw.  163.(rapid adj2 nutrition$).tw.  164.(rapid adj2 eat$).tw.  165.(rapid adj2 rehab$).tw.  166.(warm$ adj patient$).tw.  167.260. or/72-259  168. 11 and 167   1. exp Animals/ not (Humans/ and exp Animals/) 2. 168 not 169 [ Removing Animal Studies ] |
|  |
